# Avoiding Tunnel Collisions in Knee Posterolateral Corner Reconstructions: The “Versailles” Technique

**DOI:** 10.1002/atn2.70238

**Published:** 2026-08-19

**Authors:** Simon Corsia, Joseph Attas, Az‐Eddine Djebara, Philippe Boisrenoult, Nicolas Pujol

**Affiliations:** ^1^ Department of Orthopedic Surgery Centre Hospitalier de Versailles Le Chesnay France

## Abstract

The fibular collateral ligament, the popliteo‐fibular ligament and the popliteus tendon are the 3 main elements of the posterolateral corner (PLC), which is regularly injured in case of multiligament knee injuries. PLC reconstruction is recommended over repair in a large most situations, including acute cases. We describe an anatomic reconstruction of the PLC using either allografts or hamstring tendons.

## Graphical abstract

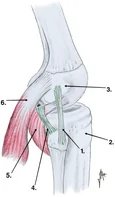

**VIDEO 1:** Lateral Versailles technique described step by step (left knee). The graft is typically prepared from a single 20‐cm tibialis anterior allograft and split in a “V” shape with one common two individual bundles, each 10‐cm long. Alternatively, other allograft or autologous hamstring tendons can be used and prepared similarly. The common strand, referred to as strand “C,” reconstructs the popliteal tendon. The 2 remaining bundles, referred to as “A” and “B,” are used separately to reconstruct the fibular collateral ligament (FCL). Each strand is tubulized with N°2 Vicryl (Ethicon, Belgium) whipstitch sutures. Incision landmarks, with the knee flexed at 90°, are the lateral epicondyle and the midpoint between the fibular head and Gerdy's tubercle. Two windows are created in the ilio‐tibial band (ITB): one for femoral exposure and one for tibial and fibular exposure. For the distal window: Posterior dissection exposes the fibular insertions of the FCL and the popliteo‐fibular ligament (PFL). For the femoral window: The ITB is incised at the level of the lateral epicondyle. Joint capsule is opened, and the femoral insertions of the FCL and the PLT are identified. The U‐shape femoral tunnel is created with the dedicated guide, by converging K‐wires at the pre‐marked insertions. Two 6‐mm tunnels are drilled. The popliteus muscle is bluntly dissected to allow direct palpation of the popliteal fossa, located approximately 10‐mm distal to the lateral tibial plateau. This corresponds to the posterior end of the tibial tunnel. A K‐wire is inserted through a stab incision at the level of Gerdy's tubercle. The tunnel is drilled from Gerdy's tubercle to the popliteal fossa, with a slightly ascending trajectory. Care is taken to avoid injury to posterior structures, with the help in this video of a custom‐made ancillary guide. A passing suture is inserted through the tunnel with the loop exiting posteriorly. The 6‐mm fibular tunnel is drilled from the FCL insertion to the PFL insertion on the head of the fibula. Its direction is anterior to posterior, distal to cranial, and lateral to medial. Two looped passing sutures are passed through the tunnel in opposite directions. With the knee at 90° of flexion, the common bundle of the graft is passed through the tibial tunnel from posterior to anterior with the passing suture The separate strands are passed under the ITB and then through the femoral tunnel from distal to proximal. Once the graft is in place, a ligament staple is used to secure the bony bridge over the femoral tunnel. The common bundle of the graft is fixed in the tibial tunnel with an interference screw without prior tapping with the knee in full extension. The separate A and B strands are then passed again under the ITB from the femur to the fibula. Using the passing sutures, the thicker strand is passed through the fibular tunnel from the FCL end to the PFL end, while the thinner strand is passed in the opposite direction. Fixation in the fibular tunnel is performed with the knee placed at 30° of flexion, neutral axis, and rotation. Tension is maintained on both strands. A 6 × 20 mm screw is inserted from distal to proximal without prior tapping. The A and B strands are tied together above the fibular head, using PDS sutures. The excess of graft emerging from the anterior exit of the fibular tunnel is also tied to the FCL. Video content can be viewed at https://doi.org/10.1002/atn2.70238.

Posterolateral corner (PLC) injuries occur in about 16% of all knee ligament injuries. LaPrade described 3 main elements.^1^ The fibular collateral ligament (FCL) acts as the main restraint to varus motion throughout the knee range of motion. The popliteus tendon (PLT) and the PFL act as secondary (Figure 1). The key role of the PLC is restriction of lateral rotation at 30° of flexion. Untreated PLC injuries lead to chronic instability and failure of other ligament reconstructions.^2^, ^3^ Identified grade‐III PLC lesions should be surgically addressed,^4^ reconstruction being preferred over repair in a large most situations.^5^ Anatomic fibular based PLC reconstructions techniques with 2 femoral tunnels are proved to be biomechanically superior.^6^ This article describes an anatomic PLC reconstruction technique.

**FIGURE 1:**
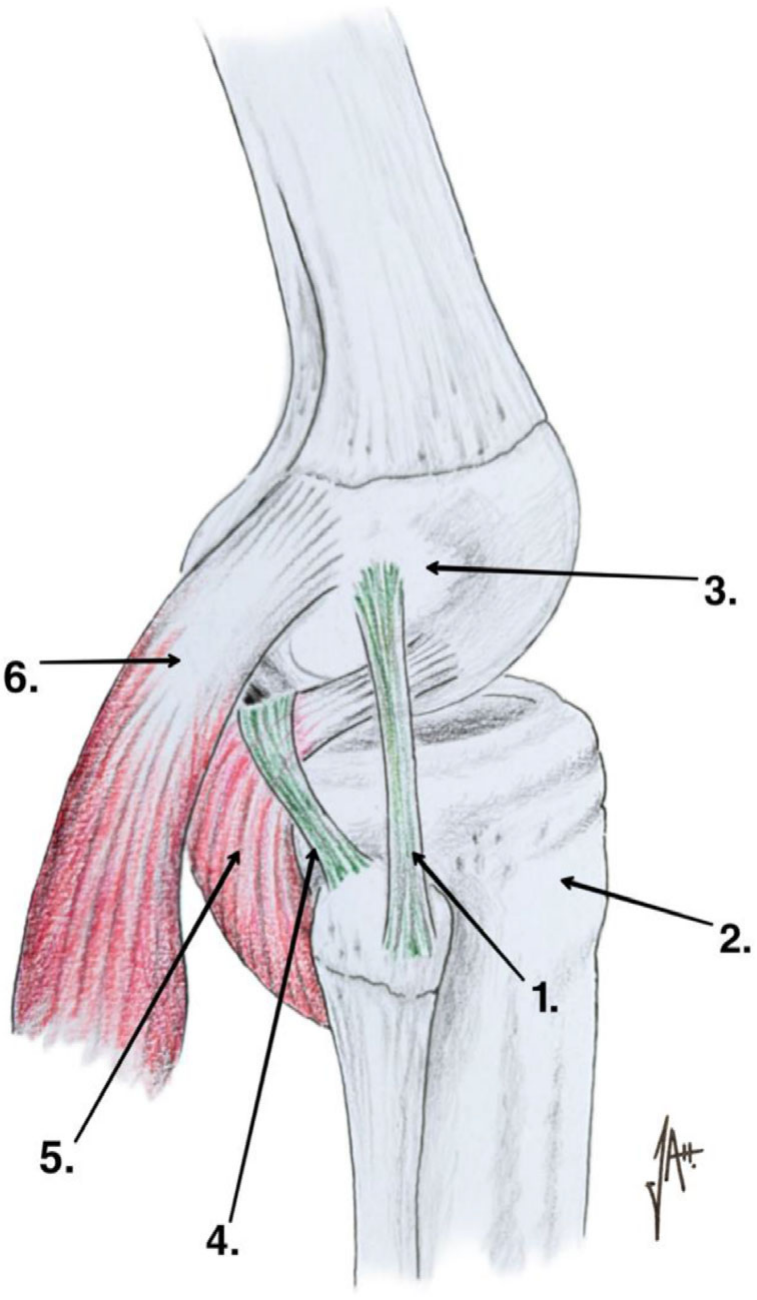
Right knee—anatomy of the posterolateral corner. (1) FCL. (2) Gerdy's tubercle is used as a palpable landmark for the tibial tunnel. (3) Lateral femoral epicondyle. (4) PFL. (5) The PLT originates at the most proximal and anterior fifth of the popliteal sulcus, at an average distance of 18.5 mm from the FCL insertion. (6) Lateral gastrocnemius muscle. (FCL, fibular collateral ligament; PFL, popliteo‐fibular ligament; PLT, popliteus muscle tendon.)

## SURGICAL TECHNIQUE

The technique is shown in Video 1. Table 1 highlights the main pearls and pitfalls of the intervention; Table 2 stresses the advantages and disadvantages of the technique.

**TABLE 1:** Technical Pearls and Pitfalls

Pearls	Pitfalls
Tubulize all 3 ends of the graft with 10‐cm long traction sutures (Vicryl 2 [Ethicon Belgium]) to help manipulate and pass the graft	Drilling femoral tunnels superior to 6.5‐mm diameter can put the bone bridge at risk
Drill the femoral tunnels with a specific aiming device. Use a Riffler to merge the tunnels	Neglecting to put a bone staple on the femoral bone bridge can lead to its fracture
Leave a strong nonabsorbable braided (author's choice: Mersuture 3 [Ethicon Belgium]) passing suture in each tunnel and secure each one with a separate clamp	Check correct placement of the fibular tunnel guide pin and do not drill over 6.5‐mm to avoid fibular head fractures
Alternatively to using suture specific suture passing instruments, arthroscopic forceps easily help drive passing sutures through bone tunnels	Drilling a fibular tunnel superior to 6.5‐mm can increase the risk of fibular head fracture
Use a staple to protect the femoral bone bridge prior to passing the graft through the tunnel	

**TABLE 2:** Advantages/Disadvantages

Advantages	Disadvantages
Avoidance of femoral tunnel collisions in multiligament knee reconstructions	Fibular nerve damage inherent to PLC reconstruction surgeries
Easier manipulation of a tendon allograft without bone blocs	Tendon allografts: increased coasts, theoretical disease transmission risk due
Anatomic fibular‐based PLC reconstructions techniques with 2 femoral tunnels are biomechanically superior	Technically demanding for free‐handed drilling of the femoral tunnel

PLC, posterolateral corner.

### Surgical Approach and Graft Preparation

Under general anesthesia, the patient is positioned supine with a thigh tourniquet except for cases with a history of initial vascular injury. The limb is placed with a lateral post and a distal foot roll with standard preparation and draping of the operating field. With the knee at 90° of flexion, a curved incision is made joining the lateral epicondyle and the midpoint between the fibular head and Gerdy's tubercle. A full thickness flap is elevated posterior to the ilio‐tibial band (ITB), exposing the biceps tendon and the fibular head. The fibular nerve is not dissected, as the surgical site is always far from it (2 cm of the proximal aspect of the fibular head only). The soleus muscle belly is bluntly elevated from the proximal tibia. The popliteal fossa can be palpated. Proximally, the ITB is incised at the level of the lateral epicondyle, and the knee capsule is opened in line to expose the femoral popliteal sulcus.

**FIGURE 2:**
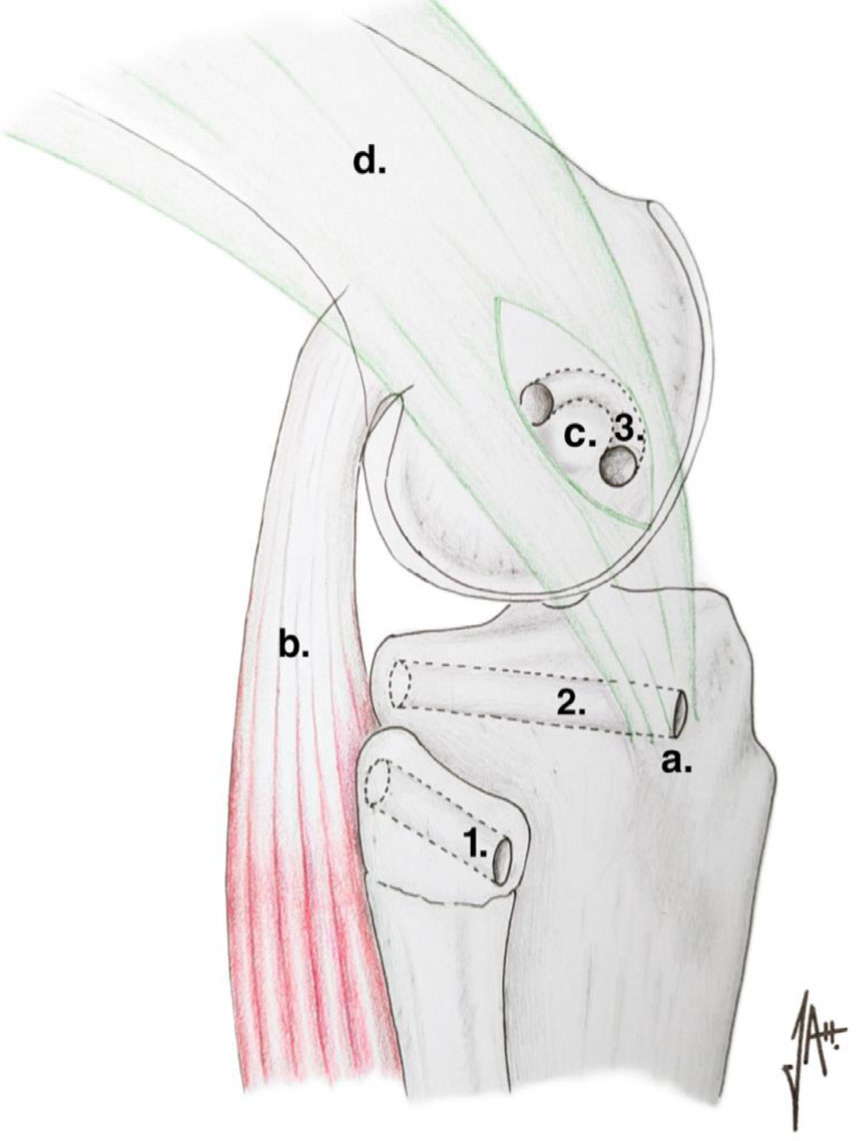
Right knee—placement of bony tunnels. (1) The fibular tunnel (6.5‐mm) is drilled from anterior to posterior, from caudal to cranial and lateral to medial, through the fibular head. The distal end of the tunnel recreates the FCL fibular insertion located on a palpable bony depression at the distal third of the lateral aspect of the fibular head; the proximal end is posteromedial down slope of the fibular head. (2) The tibial tunnel (7‐mm) is drilled from anterior to posterior through Gerdy's tubercle (a) to the popliteal fossa. (3) The FCL attaches on the femur slightly proximal and posterior to the lateral epicondyle. The PLT originates at the most proximal and anterior fifth of the popliteal sulcus, at an average distance of 18.5 mm from the FCL insertion. The drilling results in a curved femoral tunnel around the lateral femoral epicondyle (c). (b) Lateral gastrocnemius tendon. (d) Stripped ilio‐tibial band. (FCL, fibular collateral ligament; PLT, popliteus tendon.)

Transplant is typically prepared from a single 20‐cm tibialis anterior allograft. Part of the graft is divided in 2 to form a “V” shape with one common and two individual bundles, each one 10‐cm long (Figure 2). The common strand, referred to as strand “C,” reconstructs the popliteal tendon. The 2 remaining bundles, referred to as “A” and “B,” are used separately to reconstruct the FCL. Additionally, the thicker between A and B strands reconstructs the PFL. The 3 ends of each strand are prepared with N°2 Vicryl (Ethicon, Belgium) whipstitch sutures. Alternatively, other allograft or autologous hamstring tendons can be used and prepared similarly.

### Tunnel Drilling

**FIGURE 3:**
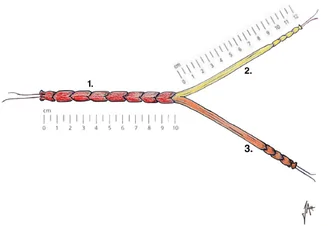
Graft preparation. The red strand is the common strand (strand C) of the graft, which will be used to reconstruct the popliteal tendon. This strand is 10 cm long. (1) Common bundle for PLT reconstruction: 10 cm length. (2) Separate strands for PFL reconstruction: at least 13 cm length. (3) Thicker of the separate strands for FCL reconstruction: at least 13 cm length. (FCL, fibular collateral ligament; PFL, popliteo‐fibular ligament; PLT, popliteus tendon.)

Tunnel locations are illustrated in Figure 3. Remnant femoral insertions of the FCL and the PLT are identified, marked and preserved. The FCL attaches slightly proximal and posterior to the lateral epicondyle. The PLT originates at the most proximal and anterior fifth of the popliteal sulcus, at an average distance of 18.5 mm from the FCL insertion.^7^ Two K‐wires are inserted from the pre‐marked insertions, converging with a 45° angle. Two blind tunnels are overdrilled using a 6.5‐mm bit. Tunnels are merged into one unique femoral arched tunnel using a Lemaire's Riffler passed from one tunnel to the other. Bony debris are cleared with saline. A passing suture (Number 2 Mersuture, Ethicon, Raritan, NJ) is driven through the tunnel and secured.

The tibial tunnel recreates the popliteus muscle insertion at its anatomic musculo‐tendinous junction, at the level of the popliteal fossa.^7^ A K‐wire is inserted from a stab incision made from a point located 0.5 cm anterior, medial, and below Gerdy's tubercle to the fossa, using an ancillary drill guide. A 7‐mm tunnel is performed. A passing stitch is driven through the tunnel and secured.

Fibular head dissection exposes the fibular insertion of the FCL and the PFL. The FCL is mainly inserted on a palpable bony depression at the distal third of the lateral aspect of the fibular head. The posterior division of the PFL attaches to the posteromedial down slope of the fibular head.^7^ A K‐wire is inserted from the FCL to the PFL insertion. A 6.5‐mm tunnel is overdrilled. Two passing sutures run through the tunnel in opposite directions in the fibular tunnel.

### Graft Passing and Fixation

**FIGURE 4:**
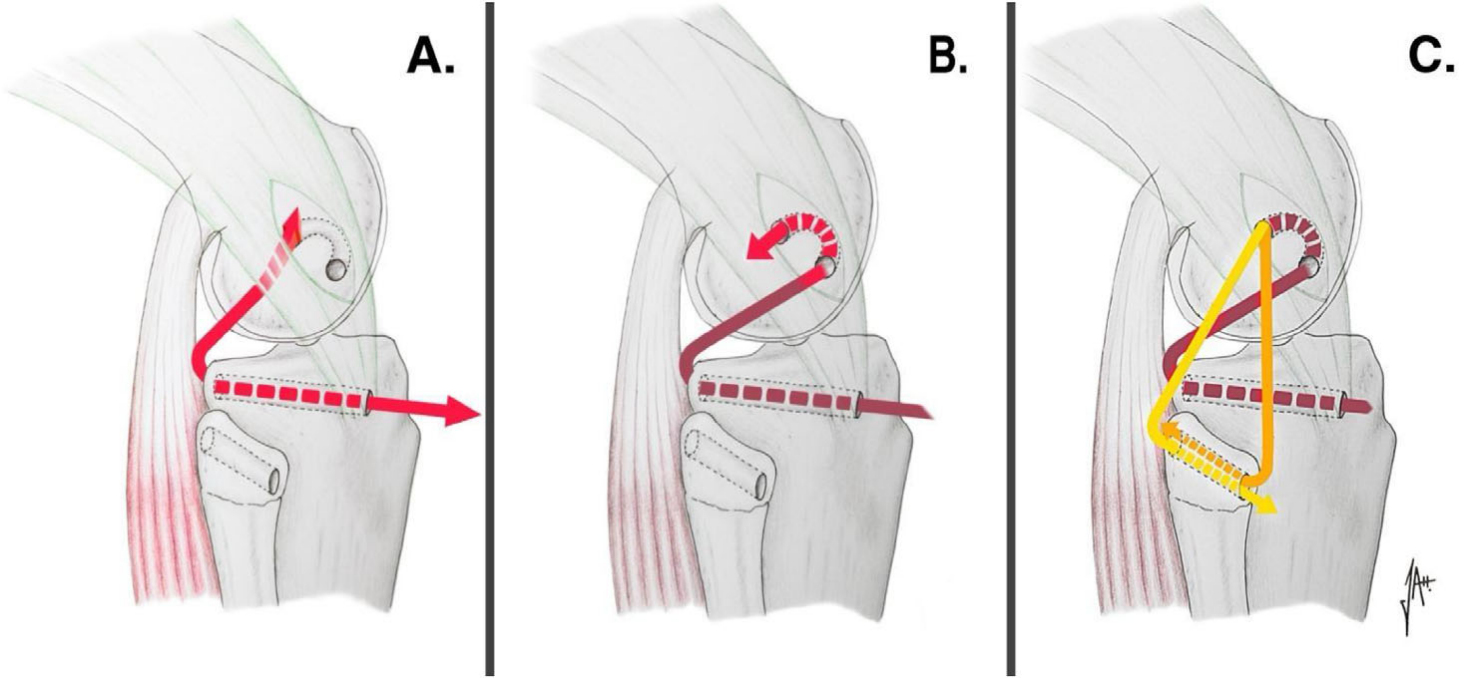
Right knee—graft passing steps. (A) First step: common bundle passed from posterior to anterior trough the tibial tunnel. Separate strands are passed under the ilio‐tibial band. (B) Second step: separate strands through the femoral tunnel from distal to proximal, then again under the ilio‐tibial band. (C) Third step: Separate strands through the fibular tunnel in opposite directions.

**FIGURE 5:**
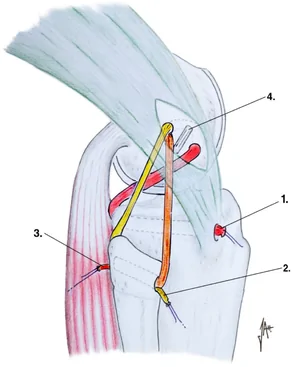
Right knee—graft fixation and final aspect. (1) Extremity of PLT strand passed from posterior to anterior through the tibial tunnel and fixed with an interference screw inserted from anterior to posterior. (2,3) Extremities of the PFL strand and the FCL strand respectively passed in opposite directions through the fibular tunnel and fixed with an interference screw inserted from anterior to posterior. (4) Blount's staple upon the bony bridge of the femoral tunnel. (FCL, fibular collateral ligament; PFL, popliteo‐fibular ligament; PLT, popliteus tendon.)

Graft is passed and fixed in a specific order (Figures 4 and 5). The knee is placed at 90° of flexion. Strand C is passed through the tibial tunnel from posterior to anterior. A clamp on the traction suture secures the graft. Strands A and B are retrieved and passed to the femur under the ITB, then through the femoral tunnel from the PLT to the FCL insertion using the passing suture. A ligament staple (Medicalex, France) is inserted over the femoral tunnel bony bridge. With the knee in full extension, strand C is fixed in the tibial tunnel using an 8 × 25‐mm bioabsorbable screw (BioSure HA/PK, Smith & Nephew). Strands A and B are passed from the femur to the fibula under the ITB. The thicker of the 2 is passed through the fibular tunnel from the FCL to the PFL insertion. The thinner strand is passed the other way. Each traction suture is individually secured. The knee is placed at 30° of flexion, neutral axis, and rotation. With the assistant pulling on each FCL strand, a 6.25 × 18‐mm (Swivelock Biotenodesis, Arthrex, Naples, FL) screw is inserted in the fibular tunnel from the FCL to PFL insertion. The portion of the graft emerging from the PFL end is sutured to the middle third of the C strand using absorbable sutures (0‐PDS II, Ethicon, France) to achieve PFL reconstruction. Strands A and B are tied together above the fibular head along with the excess of graft emerging from the FCL end of the tunnel using 0‐PDS II.

### Wound Closure and Postoperative Rehabilitation

The wound is closed in layers with a suction drain. The knee is placed in a hinged brace, for 3 months without limit of flexion. Weight bearing is initially prohibited and range of motion is restricted to 0°‐90° before full weight bearing and range of motion are allowed after 6 weeks.

## DISCUSSION

We have described the details of our technique for anatomical PLC reconstructions. This technique has been used in our department since 2002 with a first clinical series released in 2017,^8^ with other publications reported in multiligament knee injuries patients satisfying mechanical and clinical results.^9^, ^10^

Contrary to repair techniques, PLC reconstructions can address all cases.^11^, ^12^, ^13^ Anatomical techniques have proven more reliable and efficient on restoring the main PLC functions than repairs, with acknowledged biomechanical advantages.^6^

LaPrade's PLC anatomical reconstruction described in 2004, recreates the femoral insertions with 2 separate parallel tunnels. The main risk is tunnel collision especially in multiligament knee reconstruction procedures, when surgeons need to face complex intertunnels relationships.^14^, ^15^ Our technique's advantage is the arched femoral tunnel, which recreates the anatomical insertions of the PLT and FCL without the risk of tunnel convergence by drilling 2 limited length tunnels. This tunnel allows easy adjustment of the length and tensioning of the graft at final fixation, and preservation of the lateral femoral condyle bone stock. Without the need of graft fixation in the femoral tunnel, we reduce the hardware costs and risks of damaging the graft with screws. The main technical pitfall is the collapse of the lateral cortex wall between the 2 femoral tunnels. This chance is efficiently prevented with a bone staple inserted prior to tensioning the graft to reinforce the bridge. Specific drill guides are used in our department to drill the femoral and tibial tunnels. However, one can reproduce every step of the procedure using standard knee ligament devices and free‐hand K‐wire insertion.

## DISCLOSURES

The author (N.P.) declares the following financial interests/personal relationships which may be considered as potential competing interests: N.P. reports payment or honoraria for lectures, presentations, speakers bureaus, manuscript writing, or educational events from Zimmer Biomet, Smith & Nephew, Stryker: occasional consultant for education. The other authors (S.C., J.A., A‐E.D., P.B.) declare that they have no known competing financial interests or personal relationships that could have appeared to influence the work reported in this article.
